# Some fixed point results of generalized $(\phi , \psi )$-contractive mappings in ordered *b*-metric spaces

**DOI:** 10.1186/s13104-020-05354-1

**Published:** 2020-11-17

**Authors:** Belay Mitiku, Kalyani Karusala, Seshagiri Rao Namana

**Affiliations:** 1 Department of Applied Mathematics, School of Applied Natural Sciences, Adama Science and Technology University, Post Box No.1888, Adama, Ethiopia; 2 Department of Mathematics, Vignan’s Foundation for Science, Technology and Research, Vadlamudi, Andhra Pradesh 522213 India

**Keywords:** Partially ordered *b*-metric space, Fixed point, Coupled coincidence point, Coupled common fixed point, Compatible, Mixed *f*-monotone, Primary: 47H10, Secondary: 54H25

## Abstract

**Objectives:**

The aim of this paper is to establish some fixed point, coincidence point and, coupled coincidence and coupled common fixed point results for generalized $(\phi , \psi )$-contractive mappings in partially ordered *b*-metric spaces. Our results generalize, extend and unify most of the fundamental metrical fixed point theorems in the existing literature. Few examples are illustrated to justify our results.

**Result:**

The existence and uniqueness theorems for a fixed point and coincidence point, coupled coincidence point and coupled common fixed points for two mappings satisfying generalized $(\phi , \psi )$-contractive conditions in complete partially ordered *b*-metric spaces are proved. These results generalize several comparable results in the existing literature.

## Introduction and preliminaries

In analysis, the Banach contraction principle is one of the most important results and plays a crucial role in various fields of applied mathematics and scientific applications. It has been generalized and improved in many different directions. One of the most influential generalization is *b*-metric space also called metric type space by some authors, introduced and studied by Bakhtin [1] in 1989. later, Czerwik [2] generalized the Banach contraction principle in the context of complete *b*-metric spaces. After that many researchers have carried out further studies in *b*-metric space and their topological properties, some of which are in [3–18] and the references therein.

The concept of coupled fixed points for certain mappings in ordered spaces was first introduced by Bhaskar and Lakshmikantham [19] and applied their results to study the existence and uniqueness of solutions for boundary value problems. While the concept of coupled coincidence and coupled common fixed point theorems for nonlinear contractive mappings having monotone property in partially ordered complete metric spaces was first initiated by Lakshmikantham and Ćirić [20]. Since then, several authors have obtained fixed point, common fixed point, coupled coincidence point and coupled fixed point results for generalized contraction mappings in partially ordered metric spaces and partially ordered *b*-metric spaces, the readers may refer to [4, 21–42].

In this article some fixed point, coincidence, coupled coincidence and coupled common fixed points theorems are proved for mappings satisfying generalized $(\phi , \psi )$-contractions in complete partially ordered *b*-metric spaces. These results generalize and extend the results of [19, 20, 40–42] and several comparable results in the literature. Three examples are given to support our results.

The following definitions and results will be needed in what follows.

### Definition 1

[27] A map $d: P \times P \rightarrow [0, +\infty )$, where *P* is a non-empty set is said to be a *b*-metric, if it satisfies the properties given below for any $\upsilon ,\xi ,\mu \in P$ and for some real number $s \ge 1$,

- $d(\upsilon ,\xi )=0$ if and only if $\upsilon =\xi$,
- $$d(\upsilon ,\xi )=d(\xi ,\upsilon )$$,
- $$d(\upsilon ,\xi ) \le s \left( d(\upsilon ,\mu )+d(\mu ,\xi )\right) $$.

Then (*P*, *d*, *s*) is known as a *b*-metric space. If $(P,\preceq )$ is still a partially ordered set, then $(P,d,s, \preceq )$ is called a partially ordered *b*-metric space.

### Definition 2

[27] Let $(P,d,s,\preceq )$ be a *b*-metric space. Then

1. A sequence $\{\upsilon _n\}$ is said to converge to $\upsilon$, if $\lim \limits _{n \rightarrow +\infty }d(\upsilon _n,\upsilon )=0$ and written as $\lim \limits _{n \rightarrow +\infty }\upsilon _n=\upsilon$.
2. $\{\upsilon _n\}$ is said to be a Cauchy sequence in *P*, if $\lim \limits _{n,m \rightarrow +\infty }d(\upsilon _n,\upsilon _m)=0$.
3. (*P*, *d*, *s*) is said to be complete, if every Cauchy sequence in it is convergent.

### Definition 3

If the metric *d* is complete then $(P,d,s,\preceq )$ is called complete partially ordered *b*-metric space.

### Definition 4

[34] Let $(P,\preceq )$ be a partially ordered set and let $f,S:P \rightarrow P$ are two mappings. Then

1. *S* is called a monotone nondecreasing, if $S(\upsilon )\preceq S(\xi )$ for all $\upsilon ,\xi \in P$ with $\upsilon \preceq \xi$.
2. An element $\upsilon \in P$ is called a coincidence (common fixed) point of *f* and *S*, if $f\upsilon =S\upsilon \,(f\upsilon =S\upsilon =\upsilon )$.
3. *f* and *S* are called commuting, if $fS\upsilon =Sf\upsilon$, for all $\upsilon \in P$.
4. *f* and *S* are called compatible, if any sequence $\{\upsilon _n\}$ with $\lim \limits _{n \rightarrow +\infty }f\upsilon _n= \lim \limits _{n \rightarrow +\infty }S\upsilon _n =\mu ,\, \text {for}\, \mu \in P$ then $\lim \limits _{n \rightarrow +\infty } d(Sf\upsilon _n,fS\upsilon _n) =0$.
5. A pair of self maps (*f*, *S*) is called weakly compatible, if $fS\upsilon =Sf\upsilon$, when $S\upsilon =f\upsilon$ for some $\upsilon \in P$.
6. *S* is called monotone *f*-nondecreasing, if
  $$\begin{aligned} f\upsilon \preceq f\xi \Rightarrow S\upsilon \preceq S\xi ,\,\text {for any} \,\upsilon ,\xi \in P. \end{aligned}$$
7. A non empty set *P* is called well ordered set, if very two elements of it are comparable i.e., $\upsilon \preceq \xi$ or $\xi \preceq \upsilon$, for $\upsilon , \xi \in P$.

### Definition 5

[20, 26] Let $(P,d, \preceq )$ be a partially ordered set and let $S: P \times P \rightarrow P$ and $f: P \rightarrow P$ be two mappings. Then

1. *S* has the mixed *f*-monotone property, if *S* is nondecreasing *f*-monotone in its first argument and is non increasing *f*-monotone in its second argument, that is for any $\upsilon , \xi \in P$
  $$\begin{aligned} &\upsilon _1, \upsilon _2 \in P,\,\,f\upsilon _1 \preceq f\upsilon _2 \Rightarrow S(\upsilon _1,\xi )\preceq S(\upsilon _2,\xi )\, \text {and} \\&\xi _1, \xi _2 \in P,\,\,f\xi _1 \preceq f\xi _2 \Rightarrow S(\upsilon ,\xi _1)\succeq S(\upsilon ,\xi _2). \end{aligned} $$
  Suppose, if *f* is an identity mapping then *S* is said to have the mixed monotone property.
2. An element $(\upsilon ,\xi ) \in P \times P$ is called a coupled coincidence point of *S* and *f*, if $S(\upsilon ,\xi )=f\upsilon$ and $S(\xi ,\upsilon )=f\xi$. Note that, if *f* is an identity mapping then $(\upsilon ,\xi )$ is said to be a coupled fixed point of *S*.
3. Element $\upsilon \in P$ is called a common fixed point of *S* and *f*, if $S(\upsilon ,\upsilon )=f\upsilon =\upsilon$.
4. *S* and *f* are commutative, if for all $\upsilon , \xi \in P$, $S(f\upsilon ,f\xi )=f(S\upsilon ,S\xi )$.
5. *S* and *f* are said to be compatible, if
  $$\begin{aligned} \lim \limits _{n \rightarrow +\infty } d(f(S(\upsilon _n, \xi _n)), S(f\upsilon _n,f\xi _n))=0\,\text {and}\,\lim \limits _{n \rightarrow +\infty } d(f(S(\xi _n,\upsilon _n)), S(f\xi _n,f\upsilon _n))=0, \end{aligned}$$
  whenever $\{\upsilon _n\}$ and $\{\xi _n\}$ are any two sequences in *P* such that $\lim \limits _{n \rightarrow +\infty } S(\upsilon _n,\xi _n)=\lim \limits _{n \rightarrow +\infty } f\upsilon _n=\upsilon$ and $\lim \limits _{n \rightarrow +\infty } S(\xi _n,\upsilon _n)=\lim \limits _{n \rightarrow +\infty } f\xi _n=\xi$, for any $\upsilon , \xi \in P$.

The following result can be used for the convergence of a sequence in *b*-metric space.

### Lemma 6

[26] *Let* $(P,d,s,\preceq )$ *be a* *b*-*metric space with* $s>1$ *and suppose that* $\{\upsilon _n \}$ *and* $\{\xi _n\}$ *are* *b*- *convergent to* $\upsilon$ *and* $\xi$ *respectively. Then*

$$\begin{aligned} \frac{1}{s^2}d(\upsilon ,\xi )\le \lim \limits _{n \rightarrow +\infty } \inf d(\upsilon _n,\xi _n) \le \lim \limits _{n \rightarrow +\infty } \sup d(\upsilon _n,\xi _n)\le s^2 d(\upsilon ,\xi ). \end{aligned}$$

*In particular, if* $\upsilon =\xi$, *then* $\lim \limits _{n \rightarrow +\infty } d(\upsilon _n,\xi _n)=0$. *Moreover, for each* $\tau \in P$, *we have*

$$\begin{aligned} \frac{1}{s}d(\upsilon ,\tau )\le \lim \limits _{n \rightarrow +\infty } \inf d(\upsilon _n,\tau ) \le \lim \limits _{n \rightarrow +\infty } \sup d(\upsilon _n,\tau )\le s d(\upsilon ,\tau ). \end{aligned}$$

Throughout this paper, we introduce the following distance functions.

A self mapping $\phi$ defined on $[0, +\infty )$ is said to be an altering distance function, if it satisfies the following conditions:

(i) $$\phi $$ is continuous,
(ii) $\phi$ is nondecreasing,
(iii) $\phi (t)=0$ if and only if $t=0$.

Let us denote the set of all altering distance functions on $[0, +\infty )$ by $\Phi$.

Similarly, $\Psi$ denote the set of all functions $\psi :[0, +\infty )\rightarrow [0, +\infty )$ satisfying the following conditions:

(i) $\psi$ is lower semi-continuous,
(ii) $\psi (t)=0$ if and only if $t=0$.

Let $(P,d,s,\preceq )$ be a partially ordered *b*-metric space with parameter $s > 1$ and let $S:P \rightarrow P$ be a mapping. Set

1 $$\begin{aligned} M(\upsilon ,\xi )=\max \left\{ \frac{d(\xi ,S\xi ) \left[ 1+d(\upsilon ,S\upsilon )\right] }{1+d(\upsilon ,\xi )}, \frac{d(\upsilon ,S\upsilon )\,d(\xi ,S\xi )}{1+d(S\upsilon ,S\xi )}, \frac{d(\upsilon ,S\upsilon )\,d(\upsilon ,S\xi )}{1+d(\upsilon ,S\xi )+d(\xi ,S\upsilon )}, d(\upsilon ,\xi )\right\} , \end{aligned}$$

and

2 $$\begin{aligned} N(\upsilon ,\xi )=\max \left\{ \frac{d(\xi ,S\xi ) \left[ 1+d(\upsilon ,S\upsilon )\right] }{1+d(\upsilon ,\xi )}, d(\upsilon ,\xi )\right\} . \end{aligned}$$

Now, we introduced the following definition.

### Definition 7

Let $(P,d,s,\preceq )$ be a partially ordered *b*-metric space with parameter $s > 1$ and $\phi \in \Phi$, $\psi \in \Psi$. We say that $S: P \rightarrow P$ is a generalized $(\phi ,\psi )$-contractive mapping if it satisfies

3 $$\begin{aligned} \phi (sd(S\upsilon ,S\xi ))\le \phi (M(\upsilon ,\xi ))-\psi (N(\upsilon ,\xi )), \end{aligned}$$

for any $\upsilon ,\xi \in P$ with $\upsilon \preceq \xi$.

## Main text

In this section, we prove some fixed point results of mappings satisfying generalized $(\phi ,\psi )$-contraction conditions in the context of partially ordered *b*-metric spaces. The first result in this paper is the following fixed point theorem.

### Theorem 8

*Suppose that* $(P,d,s,\preceq )$ *be a complete partially ordered* *b*-*metric space with* $s > 1$. *Let* $S:P \rightarrow P$ *be an almost generalized* $(\phi ,\psi )$-*contractive mapping, and be continuous, nondecreasing mapping with regards to* $\preceq$. *If there exists* $\upsilon _0 \in P$ *with* $\upsilon _0 \preceq S\upsilon _0$, *then* *S* *has a fixed point in* *P*.

### Proof

For some $\upsilon _0 \in P$ such that $S\upsilon _0=\upsilon _0$, then we have the result. Assume that $\upsilon _0 \prec S\upsilon _0$, then construct a sequence $\{\upsilon _n\} \subset P$ by $\upsilon _{n+1}=S\upsilon _n$, for $n\ge 0$. Since *S* is nondecreasing, we obtain by induction that

4 $$\begin{aligned} \upsilon _0 \prec S\upsilon _0=\upsilon _1\preceq ....\preceq \upsilon _n \preceq S\upsilon _n=\upsilon _{n+1}\preceq .... \end{aligned}$$

If for some $n_0\in {\mathbb {N}}$ such that $\upsilon _{n_0}=\upsilon _{n_0+1}$ then from (4), $\upsilon _{n_0}$ is a fixed point of *S* and we have nothing to prove. Suppose that $\upsilon _n \ne \upsilon _{n+1}$, for all $n \ge 1$. Since $\upsilon _n>\upsilon _{n-1}$, for any $n \ge 1$ and then from contraction condition (3), we have

5 $$\begin{aligned} \begin{aligned} \phi (d(\upsilon _n,\upsilon _{n+1}))= \phi (d(S\upsilon _{n-1},S\upsilon _n))&\le \phi (sd(S\upsilon _{n-1},S\upsilon _n)) \\&\le \phi (M(\upsilon _{n-1},\upsilon _n))-\psi (N(\upsilon _{n-1},\upsilon _n)). \end{aligned} \end{aligned}$$

From (5), we get

6 $$\begin{aligned} d(\upsilon _n,\upsilon _{n+1})= d(S\upsilon _{n-1},S\upsilon _n))\le \frac{1}{s} M(\upsilon _{n-1},\upsilon _n), \end{aligned}$$

where

7 $$\begin{aligned} \begin{aligned} M(\upsilon _{n-1},\upsilon _n)&=\max \left\{ \frac{d(\upsilon _n,S\upsilon _n) \left[ 1+d(\upsilon _{n-1},S\upsilon _{n-1})\right] }{1+d(\upsilon _{n-1},\upsilon _n)}, \frac{d(\upsilon _{n-1},S\upsilon _{n-1})\,d(\upsilon _n,S\upsilon _n)}{1+d(S\upsilon _{n-1},S\upsilon _n)}, \right. \\&\quad \left. \frac{d(\upsilon _{n-1},S\upsilon _{n-1})\,d(\upsilon _{n-1},S\upsilon _n)}{1+d(\upsilon _{n-1},S\upsilon _n)+d(\upsilon _n,S\upsilon _{n-1})}, d(\upsilon _{n-1},\upsilon _n)\right\} \\&= \max \left\{ \frac{d(\upsilon _n,\upsilon _{n+1}) \left[ 1+d(\upsilon _{n-1},\upsilon _n)\right] }{1+d(\upsilon _{n-1},\upsilon _n)}, \frac{d(\upsilon _{n-1},\upsilon _n)\,d(\upsilon _n,\upsilon _{n+1})}{1+d(\upsilon _n,\upsilon _{n+1})},\right. \\&\quad \left. \frac{d(\upsilon _{n-1},\upsilon _n)\,d(\upsilon _{n-1},\upsilon _{n+1})}{1+d(\upsilon _{n-1},\upsilon _{n+1})+d(\upsilon _n,\upsilon _n)} d(\upsilon _{n-1},\upsilon _n)\right\} \\&\le \max \left\{ d(\upsilon _n,\upsilon _{n+1}), d(\upsilon _{n-1},\upsilon _n)\right\} \end{aligned} \end{aligned}$$

If $\max \{d(\upsilon _n,\upsilon _{n+1}),d(\upsilon _{n-1}, \upsilon _n)\}= d(\upsilon _n,\upsilon _{n+1})$ for some $n \ge 1$, then from (6) follows

8 $$\begin{aligned} d(\upsilon _n,\upsilon _{n+1})\le \frac{1}{s} d(\upsilon _n,\upsilon _{n+1}), \end{aligned}$$

which is a contradiction. This means that $\max \{d(\upsilon _n,\upsilon _{n+1}),d(\upsilon _{n-1},\upsilon _n)\}= d(\upsilon _{n-1},\upsilon _n)$ for $n \ge 1$. Hence, we obtain from (6) that

9 $$\begin{aligned} d(\upsilon _n,\upsilon _{n+1})\le \frac{1}{s} d(\upsilon _{n-1},\upsilon _n). \end{aligned}$$

Since, $\frac{1}{s}\in (0,1)$ then the sequence $\{\upsilon _n\}$ is a Cauchy sequence by [3, 5, 8, 10]. Since *P* is complete, then there exists some $\mu \in P$ such that $\upsilon _n \rightarrow \mu$.

Further, the continuity of *S* implies that

10 $$\begin{aligned} S\mu =S(\lim \limits _{n\rightarrow +\infty }\upsilon _n)=\lim \limits _{n\rightarrow +\infty }S\upsilon _n=\lim \limits _{n\rightarrow +\infty }\upsilon _{n+1}=\mu . \end{aligned}$$

Therefore, $\mu$ is a fixed point of *S* in *P*. $\square$

Last result is still valid for *S* not necessarily continuous, assuming an additional hypothesis on *P*.

### Theorem 9

*Let* $(P,d,s,\preceq )$ *be a complete partially ordered* *b*-*metric space with* $s > 1$. *Assume that* *P* *satisfies*

$$\begin{aligned} \text {if a nondecreasing sequence}\, \{\upsilon _n\} \rightarrow \mu \, \text {in}\, P,\, \text {then}\, \upsilon _n \preceq \mu \, \text {for all}\, n \in {\mathbb {N}},\,\text {i.e.,}\, \mu =\sup \upsilon _n. \end{aligned}$$

*Let* $S:P \rightarrow P$ *be a nondecreasing mapping such that the contraction condition* (3) *is satisfied. If there exists* $\upsilon _0 \in P$ *with* $\upsilon _0 \preceq S\upsilon _0$, *then* *S* *has a fixed point in* *P*.

### Proof

From the proof of Theorem 8, we construct a nondecreasing Cauchy sequence $\{\upsilon _n\}$, which converges to $\mu$ in *P*. Therefore from the hypotheses, we have $\upsilon _n \preceq \mu$ for any $n \in {\mathbb {N}}$, implies that $\mu =\sup \upsilon _n$.

Now, we prove that $\mu$ is a fixed point of *S*, that is $S\mu =\mu$. Suppose that $S\mu \ne \mu$. Let

11 $$\begin{aligned}  M(\upsilon _n,\mu )&=\max \left\{ \frac{d(\mu ,S\mu ) \left[ 1+d(\upsilon _n,S\upsilon _n)\right] }{1+d(\upsilon _n,\mu )}, \frac{d(\upsilon _n,S\upsilon _n)\,d(\mu ,S\mu )}{1+d(S\upsilon _n,S\mu )},\right.\\&\quad \left. \frac{d(\upsilon _n,S\upsilon _n)\,d(\upsilon _n,S\mu )}{1+d(\upsilon _n,S\mu )+d(\mu ,S\upsilon _n)}, d(\upsilon _n,\mu )\right\}, \end{aligned} $$

and

12 $$\begin{aligned} N(\upsilon _n,\mu )=\max \{\frac{d(\mu ,S\mu ) \left[ 1+d(\upsilon _n,S\upsilon _n)\right] }{1+d(\upsilon _n,\mu )}, d(\upsilon _n,\mu )\}. \end{aligned}$$

Letting $n\rightarrow +\infty$ and from the fact that $\lim \limits _{n\rightarrow +\infty }\upsilon _n=\mu$, we get

13 $$\begin{aligned} \lim \limits _{n \rightarrow +\infty }M(\upsilon _n, \mu )= \max \{d(\mu ,S\mu ),0,0,0\}=d(\mu ,S\mu ), \end{aligned}$$

and

14 $$\begin{aligned} \lim \limits _{n \rightarrow +\infty }N(\upsilon _n, \mu )= \max \{d(\mu ,S\mu ),0\}=d(\mu ,S\mu ). \end{aligned}$$

We know that $\upsilon _n \preceq \mu$, for all *n* then from contraction condition (3), we get

15 $$\begin{aligned} \phi (d(\upsilon _{n+1}, S\mu ))=\phi (d(S\upsilon _n, S\mu )\le \phi (s d(S\upsilon _n, S\mu )\le \phi (M(\upsilon _n, \mu ))-\psi (N(\upsilon _n, \mu )). \end{aligned}$$

Letting $n \rightarrow +\infty$ and use of (13) and (14), we get

16 $$\begin{aligned} \phi (d(\mu ,S\mu )) \le \phi (d(\mu ,S\mu ))-\psi (d(\mu ,S\mu ))< \phi (d(\mu ,S\mu )), \end{aligned}$$

which is a contradiction under (16). Thus, $S\mu =\mu$, that is *S* has a fixed point $\mu$ in *P*. $\square$

Now we give the sufficient condition for the uniqueness of the fixed point exists in Theorems 8 and 9.

17 $$\begin{aligned} \text {every pair of elements has a lower bound or an upper bound.} \end{aligned}$$

This condition is equivalent to,

$$\begin{aligned} \text {for every}\, \upsilon , \xi \in P, \,\text {there exists}\, w \in P \,\text {which is comparable to}\, \upsilon \, \text {and}\, \xi . \end{aligned}$$

### Theorem 10

*In addition to the hypotheses of Theorem* 8*(or Theorem*9), *condition* (17) *provides uniqueness of the fixed point of* *S* *in* *P*.

### Proof

From Theorem 8 (or Theorem 9), we conclude that *S* has a nonempty set of fixed points. Suppose that $\upsilon ^*$ and $\xi ^*$ be two fixed points of *S* then, we claim that $\upsilon ^*=\xi ^*$. Suppose that $\upsilon ^*\ne \xi ^*$, then from the hypotheses we have

18 $$\begin{aligned} \begin{aligned} \phi (d(S\upsilon ^*, S\xi ^*))&\le \phi (sd(S\upsilon ^*, S\xi ^*)) \le \phi (M(\upsilon ^*, \xi ^*))-\psi (N(\upsilon ^*, \xi ^*)). \end{aligned} \end{aligned}$$

Consequently, we get

19 $$\begin{aligned} d(\upsilon ^*, \xi ^*))= d(S\upsilon ^*, S\xi ^*)) \le \frac{1}{s} M(\upsilon ^*, \xi ^*), \end{aligned}$$

where

20 $$\begin{aligned} \begin{aligned} M(\upsilon ^*,\xi ^*)&=\max \left\{ \frac{d(\xi ^*,S\xi ^*) \left[ 1+d(\upsilon ^*,S\upsilon ^*)\right] }{1+d(\upsilon ^*,\xi ^*)}, \frac{d(\upsilon ^*,S\upsilon ^*)\,d(\xi ^*,S\xi ^*)}{1+d(S\upsilon ^*,S\xi ^*)}, \right. \\&\quad \left. \frac{d(\upsilon ^*,S\upsilon ^*)\,d(\upsilon ^*,S\xi ^*)}{1+d(\upsilon ^*,S\xi ^*)+d(\xi ^*,S\upsilon ^*)}, d(\upsilon ^*,\xi ^*)\right\} \\&=\max \left\{ \frac{d(\xi ^*,\xi ^*) \left[ 1+d(\upsilon ^*,\upsilon ^*)\right] }{1+d(\upsilon ^*,\xi ^*)}, \frac{d(\upsilon ^*,\upsilon ^*)\,d(\xi ^*,\xi ^*)}{1+d(\upsilon ^*,\xi ^*)}, \frac{d(\upsilon ^*,\upsilon ^*)\,d(\upsilon ^*,\xi ^*)}{1+d(\upsilon ^*,\xi ^*)+d(\xi ^*,\upsilon ^*)},\right. \\&\quad d(\upsilon ^*,\xi ^*)\} \\&= \max \{0,0,0, d(\upsilon ^*,\xi ^*) \} \\&=d(\upsilon ^*,\xi ^*) \end{aligned} \end{aligned}$$

From (19), we obtain that

21 $$\begin{aligned} d(\upsilon ^*, \xi ^*) \le \frac{1}{s} d(\upsilon ^*, \xi ^*)<d(\upsilon ^*, \xi ^*), \end{aligned}$$

which is a contradiction. Hence, $\upsilon ^*= \xi ^*$. This completes the proof. $\square$

Let $(P,d,s,\preceq )$ be a partially ordered *b*-metric space with parameter $s > 1$, and let $S,f:P \rightarrow P$ be two mappings. Set

22 $$\begin{aligned} \begin{aligned} M_f(\upsilon ,\xi )&=\max \left\{ \frac{d(f\xi ,S\xi ) \left[ 1+d(f\upsilon ,S\upsilon )\right] }{1+d(f\upsilon ,f\xi )}, \frac{d(f\upsilon ,S\upsilon )\,d(f\xi ,S\xi )}{1+d(S\upsilon ,S\xi )},\right. \\&\quad \left. \frac{d(f\upsilon ,S\upsilon )\,d(f\upsilon ,S\xi )}{1+d(f\upsilon ,S\xi )+d(f\xi ,S\upsilon )}, d(f\upsilon ,f\xi )\right\} , \end{aligned} \end{aligned}$$

and

23 $$\begin{aligned} N_f(\upsilon ,\xi )=\max \left\{ \frac{d(f\xi ,S\xi ) \left[ 1+d(f\upsilon ,S\upsilon )\right] }{1+d(f\upsilon ,f\xi )}, d(f\upsilon ,f\xi )\right\} . \end{aligned}$$

Now, we introduce the following definition.

### Definition 11

Let $(P,d,s,\preceq )$ be a partially ordered *b*-metric space with parameter $s > 1$. The mapping $S:P \rightarrow P$ is called a generalized $(\phi ,\psi )$-contraction mapping with respect to $f:P \rightarrow P$ for some $\phi \in \Phi$ and $\psi \in \Psi$, if

24 $$\begin{aligned} \phi (sd(S\upsilon ,S\xi ))\le \phi (M_f(\upsilon ,\xi ))-\psi (N_f(\upsilon ,\xi )), \end{aligned}$$

for any $\upsilon ,\xi \in P$ with $f\upsilon \preceq f\xi$, where $M_f(\upsilon ,\xi )$ and $N_f(\upsilon ,\xi )$ are given by (22) and (23) respectively.

### Theorem 12

*Suppose that* $(P,d,s,\preceq )$ *be a complete partially ordered* *b*-*metric space with* $s> 1$. *Let* $S: P \rightarrow P$ *be an almost generalized* $(\phi ,\psi )$-*contractive mapping with respect to* $f: P \rightarrow P$ *and*, *S* *and* *f* *are continuous such that* *S* *is a monotone* *f*-*non decreasing mapping, compatible with* *f* *and* $SP \subseteq fP$. *If for some* $\upsilon _0 \in P$ *such that* $f\upsilon _0 \preceq S\upsilon _0$, *then* *S* *and* *f* *have a coincidence point in* *P*.

### Proof

By following the proof of a Theorem 2.2 in [30], we construct two sequences $\{\upsilon _n\}$ and $\{\xi _n\}$ in *P* such that

25 $$\begin{aligned} \xi _n=S\upsilon _n=f\upsilon _{n+1} \,\text {for all}\,n\ge 0, \end{aligned}$$

for which

26 $$\begin{aligned} f\upsilon _0 \preceq f\upsilon _1 \preceq .... \preceq f\upsilon _n \preceq f\upsilon _{n+1} \preceq ..... \end{aligned}$$

Again from [30], we have to show that

27 $$\begin{aligned} d(\xi _n,\xi _{n+1})\le \lambda d(\xi _{n-1},\xi _n), \end{aligned}$$

for all $n \ge 1$ and where $\lambda \in [0, \frac{1}{s})$. Now from (24) and use of (25) and (26), we have

28 $$\begin{aligned} \begin{aligned} \phi (sd(\xi _n,\xi _{n+1}))&=\phi (sd(S\upsilon _n,S\upsilon _{n+1}))\\&\le \phi (M_f(\upsilon _n,\upsilon _{n+1}))-\psi (N_f(\upsilon _n,\upsilon _{n+1})), \end{aligned} \end{aligned}$$

where

$$\begin{aligned} \begin{aligned} M_f(\upsilon _n,\upsilon _{n+1})&=\max \left\{ \frac{d(f\upsilon _{n+1},S\upsilon _{n+1}) \left[ 1+d(f\upsilon _n,S\upsilon _n)\right] }{1+d(f\upsilon _n,f\upsilon _{n+1})}, \frac{d(f\upsilon _n,S\upsilon _n)\,d(f\upsilon _{n+1},S\upsilon _{n+1})}{1+d(S\upsilon _n,S\upsilon _{n+1})},\right. \\&\quad \left. \frac{d(f\upsilon _n,S\upsilon _n)\,d(f\upsilon _n,S\upsilon _{n+1})}{1+d(f\upsilon _n,S\upsilon _{n+1})+d(f\upsilon _{n+1},S\upsilon _n)}, d(f\upsilon _n,f\upsilon _{n+1})\right\} \\&=\max \left\{ \frac{d(\xi _n,\xi _{n+1}) \left[ 1+d(\xi _{n-1},\xi _n)\right] }{1+d(\xi _{n-1},\xi _n)}, \frac{d(\xi _{n-1},\xi _n)\,d(\xi _n,\xi _{n+1})}{1+d(\xi _n,\xi _{n+1})},\right. \\&\quad \left. \frac{d(\xi _{n-1},\xi _n)\,d(\xi _{n-1},\xi _{n+1})}{1+d(\xi _{n-1},\xi _{n+1})+d(\xi _n,\xi _n)},d(\xi _{n-1},\xi _n)\right\} \\&\le \max \left\{ d(\xi _{n-1},\xi _n),d(\xi _n,\xi _{n+1})\right\} \end{aligned} \end{aligned}$$

and

$$\begin{aligned} \begin{aligned} N_f(\upsilon _n,\upsilon _{n+1})&=\max \left\{ \frac{d(f\upsilon _{n+1},S\upsilon _{n+1}) \left[ 1+d(f\upsilon _n,S\upsilon _n)\right] }{1+d(f\upsilon _n,f\upsilon _{n+1})},d(f\upsilon _n,f\upsilon _{n+1})\right\} \\&=\max \left\{ \frac{d(\xi _n,\xi _{n+1}) \left[ 1+d(\xi _{n-1},\xi _n)\right] }{1+d(\xi _{n-1},\xi _n)},d(\xi _{n-1},\xi _n)\right\} \\&=\max \{d(\xi _{n-1},\xi _n),d(\xi _n,\xi _{n+1})\} \end{aligned} \end{aligned}$$

Therefore from equation (28), we get

29 $$\begin{aligned} \phi (sd(\xi _n,\xi _{n+1}))\le \phi (\max \{d(\xi _{n-1},\xi _n),d(\xi _n,\xi _{n+1})\})-\psi (\max \{d(\xi _{n-1},\xi _n),d(\xi _n,\xi _{n+1})\}). \end{aligned}$$

If $0<d(\xi _{n-1},\xi _n)\le d(\xi _n,\xi _{n+1})$ for some $n \in {\mathbb {N}}$, then from (29) we get

30 $$\begin{aligned} \phi (sd(\xi _n,\xi _{n+1}))\le \phi (d(\xi _n,\xi _{n+1}))-\psi (d(\xi _n,\xi _{n+1}))<\phi (d(\xi _n,\xi _{n+1})), \end{aligned}$$

or equivalently

31 $$\begin{aligned} sd(\xi _n,\xi _{n+1})\le d(\xi _n,\xi _{n+1}). \end{aligned}$$

This is a contradiction. Hence from (29) we obtain that

32 $$\begin{aligned} sd(\xi _n,\xi _{n+1})\le d(\xi _{n-1},\xi _n). \end{aligned}$$

Thus equation (27) holds, where $\lambda \in [0,\frac{1}{s})$. Therefore from (27) and Lemma 3.1 of [16], we conclude that $\{\xi _n\}=\{S\upsilon _n\}=\{f\upsilon _{n+1}\}$ is a Cauchy sequence in *P* and then converges to some $\mu \in P$ as *P* is complete such that

$$\begin{aligned} \lim \limits _{n \rightarrow +\infty }S\upsilon _{n}=\lim \limits _{n \rightarrow +\infty }f\upsilon _{n+1}=\mu . \end{aligned}$$

Thus by the compatibility of *S* and *f*, we obtain that

33 $$\begin{aligned} \lim \limits _{n \rightarrow +\infty }d(f(S\upsilon _n), S(f\upsilon _n))=0, \end{aligned}$$

and from the continuity of *S* and *f*, we have

34 $$\begin{aligned} \lim \limits _{n \rightarrow +\infty }f(S\upsilon _n)=f\mu ,\,\,\,\,\,\,\,\,\,\,\,\lim \limits _{n \rightarrow +\infty } S(f\upsilon _n)=S\mu . \end{aligned}$$

Further by use of triangular inequality and from equations (33) and (34) , we get

35 $$\begin{aligned} \frac{1}{s}d(S\mu ,f\mu )\le d(S\mu ,S(f\upsilon _n))+s d(S(f\upsilon _n), f(S\upsilon _n))+sd(f(S\upsilon _n), f\mu ). \end{aligned}$$

Finally, we arrive at $d(Sv,fv)=0$ as $n \rightarrow +\infty$ in (35). Therefore, *v* is a coincidence point of *S* and *f* in *P*. $\square$

Relaxing the continuity of *f* and *S* in Theorem 12, we obtain the following result.

### Theorem 13

*In Theorem* 12, *assume that* *P* *satisfies*

$$\begin{aligned} &\text { for any nondecreasing sequence}\, \{f\upsilon _n\}\subset P\,\text {with}\, \lim \limits _{n \rightarrow +\infty } f\upsilon _n=f\upsilon \, \text {in}\, fP,\, \text {where}\,fP \\&\text {is a closed subset of}\, P\,\text {implies that}\, f\upsilon _n \preceq f\upsilon , f\upsilon \preceq f(f\upsilon )\,\text {for}\,n \in {\mathbb {N}}. \end{aligned}$$

*If there exists* $\upsilon _0 \in P$ *such that* $f\upsilon _0 \preceq S\upsilon _0$, *then the weakly compatible mappings* *S* *and* *f* *have a coincidence point in* *P*. *Moreover*, *S* *and* *f* *have a common fixed point, if* *S* *and* *f* *commute at their coincidence points.*

### Proof

The sequence, $\{\xi _n\}=\{S\upsilon _n\}=\{f\upsilon _{n+1}\}$ is a Cauchy sequence from the proof of Theorem 12. Since *fP* is closed, then there is some $\mu \in P$ such that

$$\begin{aligned} \lim \limits _{n \rightarrow +\infty }S\upsilon _{n}=\lim \limits _{n \rightarrow +\infty }f\upsilon _{n+1}=f\mu . \end{aligned}$$

Thus by the hypotheses, we have $f\upsilon _n\preceq f\mu$ for all $n \in {\mathbb {N}}$. Now, we have to prove that $\mu$ is a coincidence point of *S* and *f*.

From equation (24), we have

36 $$\begin{aligned} \begin{aligned} \phi (sd(S\upsilon _n,S\upsilon ))\le \phi (M_f(\upsilon _n,\upsilon ))-\psi (N_f(\upsilon _n,\upsilon )), \end{aligned} \end{aligned}$$

where

$$\begin{aligned} \begin{aligned} M_f(\upsilon _n,\mu )&=\max \left\{ \frac{d(f\mu ,S\mu ) \left[ 1+d(f\upsilon _n,S\upsilon _n)\right] }{1+d(f\upsilon _n,f\mu )}, \frac{d(f\upsilon _n,S\upsilon _n)\,d(f\mu ,S\mu )}{1+d(S\upsilon _n,S\mu )},\right. \\&\quad \left. \frac{d(f\upsilon _n,S\upsilon _n)\,d(f\upsilon _n,S\mu )}{1+d(f\upsilon _n,S\mu )+d(f\mu ,S\upsilon _n)}, d(f\upsilon _n,f\mu )\right\} \\&\quad \rightarrow \max \{d(f\mu ,S\mu ),0,0,0\} \\&= d(f\mu ,S\mu ) \,\text {as}\,n \rightarrow +\infty , \end{aligned} \end{aligned}$$

and

$$\begin{aligned} \begin{aligned} N_f(\upsilon _n,\mu )&=\max \left\{ \frac{d(f\mu ,S\mu ) \left[ 1+d(f\upsilon _n,S\upsilon _n)\right] }{1+d(f\upsilon _n,f\mu )},d(f\upsilon _n,f\mu )\} \right\}&\rightarrow \max \left\{ d(f\mu ,S\mu ),0\} \right\}&= d(f\mu ,S\mu ) \,\text {as}\,n \rightarrow +\infty . \end{aligned} \end{aligned}$$

Therefore equation (36) becomes

37 $$\begin{aligned} \phi (s\lim \limits _{n \rightarrow +\infty } d(S\upsilon _n,S\upsilon ))\le \phi (d(f\mu ,S\mu ))-\psi (d(f\mu ,S\mu ))< \phi (d(f\mu ,S\mu )). \end{aligned}$$

Consequently, we get

38 $$\begin{aligned} \lim \limits _{n \rightarrow +\infty }d(S\upsilon _n,S\upsilon ) < \frac{1}{s}d(f\mu ,S\mu ). \end{aligned}$$

Further by triangular inequality, we have

39 $$\begin{aligned} \frac{1}{s}d(f\mu ,S\mu )\le d(f\mu ,S\upsilon _n)+d(S\upsilon _n,S\mu ), \end{aligned}$$

then (38) and (39) lead to contradiction, if $f\mu \ne S\mu$. Hence, $f\mu =S\mu$. Let $f\mu =S\mu =\rho$, that is *S* and *f* commute at $\rho$, then $S\rho = S(f\mu )=f(S\mu )=f\rho$. Since $f\mu =f(f\mu )=f\rho$, then by equation (36) with $f\mu =S\mu$ and $f\rho =S\rho$, we get

40 $$\begin{aligned} \begin{aligned} \phi (sd(S\mu ,S\rho ))\le \phi (M_f(\mu ,\rho ))-\psi (N_f(\mu ,\rho ))<\phi (d(S\mu ,S\rho )), \end{aligned} \end{aligned}$$

or equivalently,

$$\begin{aligned} sd(S\mu ,S\rho ) \le d(S\mu ,S\rho ), \end{aligned}$$

which is a contradiction, if $S\mu \ne S\rho$. Thus, $S\mu = S\rho = \rho$. Hence, $S\mu = f\rho =\rho$, that is $\rho$ is a common fixed point of *S* and *f*. $\square$

### Definition 14

Let $(P,d,s,\preceq )$ be a complete partially ordered *b*-metric space with $s > 1$, $\phi \in \Phi$ and $\psi \in \Psi$. A mapping $S:P \times P \rightarrow P$ is said to be an almost generalized $(\phi ,\psi )$-contractive mapping with respect to $f:P \rightarrow P$ such that

41 $$\begin{aligned} \phi (s^kd(S(\upsilon ,\xi ),S(\rho ,\tau )))\le \phi (M(\upsilon ,\xi ,\rho ,\tau ))-\psi (N(\upsilon ,\xi ,\rho ,\tau )), \end{aligned}$$

for all $\upsilon ,\xi ,\rho ,\tau \in P$ with $f\upsilon \preceq f \rho$ and $f\xi \succeq f \tau$, $k>2$ where

42 $$\begin{aligned} \begin{aligned} M_f(\upsilon ,\xi ,\rho ,\tau )&=\max \left\{ \frac{d(f\rho ,S(\rho , \tau )) \left[ 1+d(f\upsilon ,S(\upsilon , \xi ))\right] }{1+d(f\upsilon ,f\rho )}, \frac{d(f\upsilon ,S(\upsilon , \xi ))\,d(f\rho ,S(\rho , \tau ))}{1+d(S(\upsilon , \xi ),S(\rho , \tau ))},\right. \\&\quad \left. \frac{d(f\upsilon ,S(\upsilon , \xi ))\,d(f\upsilon ,S(\rho , \tau ))}{1+d(f\upsilon ,S(\rho , \tau ))+d(f\rho ,S(\upsilon , \xi ))}, d(f\upsilon ,f\rho )\right\} , \end{aligned} \end{aligned}$$

and

$$\begin{aligned} N_f(\upsilon ,\xi ,\rho ,\tau )=\max \left\{ \frac{d(f\rho ,S(\rho ,\tau )) \left[ 1+d(f\upsilon ,S(\upsilon ,\xi ))\right] }{1+d(f\upsilon ,f\rho )}, d(f\upsilon ,f\rho )\right\} . \end{aligned}$$

### Theorem 15

*Let* $(P,d,s,\preceq )$ *be a complete partially ordered* *b*-*metric space with* $s > 1$. *Suppose that* $S:P \times P \rightarrow P$ *be an almost generalized* $(\phi ,\psi )$-*contractive mapping with respect to* $f:P \rightarrow P$ *and*, *S* *and* *f* *are continuous functions such that* *S* *has the mixed* *f*-*monotone property and commutes with* *f*. *Also assume that* $S(P \times P) \subseteq f(P)$. *Then* *S* *and* *f* *have a coupled* *coincidence point in* *P*, *if there exists* $(\upsilon _0,\xi _0) \in P \times P$ *such that* $f\upsilon _0 \preceq S(\upsilon _0,\xi _0)$ *and* $f\xi _0 \succeq S(\xi _0,\upsilon _0)$.

### Proof

From the hypotheses and following the proof of Theorem 2.2 of [30], we construct two sequences $\{\upsilon _n\}$ and $\{\xi _n\}$ in *P* such that

$$\begin{aligned} f\upsilon _{n+1}=S(\upsilon _n,\xi _n), \,\,\,\,f\xi _{n+1}=S(\xi _n,\upsilon _n),\,\,\text {for all}\,n\ge 0. \end{aligned}$$

In particular, $\{f\upsilon _n\}$ is nondecreasing and $\{f\xi _n\}$ is nonincreasing sequences in *P*. Now from (41) by replacing $\upsilon =\upsilon _n, \xi =\xi _n, \rho =\upsilon _{n+1}, \tau =\xi _{n+1}$, we get

43 $$\begin{aligned} \begin{aligned} \phi (s^kd(f\upsilon _{n+1},f\upsilon _{n+2}))&=\phi (s^kd(S(\upsilon _n,\xi _n),S(\upsilon _{n+1},\xi _{n+1})))\\&\le \phi (M_f(\upsilon _n,\xi _n,\upsilon _{n+1},\xi _{n+1}))-\psi (N_f(\upsilon _n,\xi _n,\upsilon _{n+1},\xi _{n+1})), \end{aligned} \end{aligned}$$

where

44 $$\begin{aligned} M_f(\upsilon _n,\xi _n,\upsilon _{n+1},\xi _{n+1})\le \max \{d(f\upsilon _n,f\upsilon _{n+1}),d(f\upsilon _{n+1},f\upsilon _{n+2})\} \end{aligned}$$

and

45 $$\begin{aligned} N_f(\upsilon _n,\xi _n,\upsilon _{n+1},\xi _{n+1})= \max \{d(f\upsilon _n,f\upsilon _{n+1}),d(f\upsilon _{n+1},f\upsilon _{n+2})\}. \end{aligned}$$

Therefore from (43), we have

46 $$\begin{aligned} \begin{aligned} \phi (s^kd(f\upsilon _{n+1},f\upsilon _{n+2}))&\le \phi (\max \{d(f\upsilon _n,f\upsilon _{n+1}),d(f\upsilon _{n+1},f\upsilon _{n+2})\})\\&\quad -\psi (\max \{d(f\upsilon _n,f\upsilon _{n+1}),d(f\upsilon _{n+1},f\upsilon _{n+2})\}). \end{aligned} \end{aligned}$$

Similarly by taking $\upsilon =\xi _{n+1}, \xi =\upsilon _{n+1}, \rho =\upsilon _n, \tau =\upsilon _n$ in (41), we get

47 $$\begin{aligned} \begin{aligned} \phi (s^kd(f\xi _{n+1},f\xi _{n+2}))&\le \phi (\max \{d(f\xi _n,f\xi _{n+1}),d(f\xi _{n+1},f\xi _{n+2})\})\\&\quad -\psi (\max \{d(f\xi _n,f\xi _{n+1}),d(f\xi _{n+1},f\xi _{n+2})\}). \end{aligned} \end{aligned}$$

From the fact that $\max \{\phi (c),\phi (d)\}=\phi \{\max \{c,d\}\}$ for all $c,d \in [0,+\infty )$. Then combining (46) and (47), we get

48 $$\begin{aligned} \begin{aligned} \phi (s^k \delta _n)&\le \phi (\max \{d(f\upsilon _n,f\upsilon _{n+1}),d(f\upsilon _{n+1},f\upsilon _{n+2}),d(f\xi _n,f\xi _{n+1}),d(f\xi _{n+1},f\xi _{n+2})\})\\&\quad -\psi (\max \{d(f\upsilon _n,f\upsilon _{n+1}),d(f\upsilon _{n+1},f\upsilon _{n+2}),d(f\xi _n,f\xi _{n+1}),d(f\xi _{n+1},f\xi _{n+2})\}), \end{aligned} \end{aligned}$$

where

49 $$\begin{aligned} \delta _n=\max \{d(f\upsilon _{n+1},f\upsilon _{n+2}), d(f\xi _{n+1},f\xi _{n+2})\}. \end{aligned}$$

Let us denote,

50 $$\begin{aligned} \Delta _n=\max \{d(f\upsilon _n,f\upsilon _{n+1}),d(f\upsilon _{n+1},f\upsilon _{n+2}),d(f\xi _n,f\xi _{n+1}),d(f\xi _{n+1},f\xi _{n+2})\}. \end{aligned}$$

Hence from equations (46)-(49), we obtain

51 $$\begin{aligned} s^k\delta _n\le \Delta _n. \end{aligned}$$

Next, we prove that

52 $$\begin{aligned} \delta _n\le \lambda \delta _{n-1}, \end{aligned}$$

for all $n \ge 1$ and where $\lambda =\frac{1}{s^k} \in [0,1)$.

Suppose that if $\Delta _n=\delta _n$ then from (51), we get $s^k\delta _n\le \delta _n$ which leads to $\delta _n=0$ as $s>1$ and hence (52) holds. If $\Delta _n=\max \{d(f\upsilon _n,f\upsilon _{n+1}), d(f\xi _n,f\xi _{n+1})\}$, i.e., $\Delta _n=\delta _{n-1}$ then (51) follows (52).

Now from (51), we obtain that $\delta _n\le \lambda ^n \delta _0$ and hence,

53 $$\begin{aligned} d(f\upsilon _{n+1},f\upsilon _{n+2})\le \lambda ^n \delta _0 \,\,\text {and}\,\,d(f\xi _{n+1},f\xi _{n+2})\le \lambda ^n \delta _0. \end{aligned}$$

Therefore from Lemma 3.1 of [16], the sequences $\{f\upsilon _n\}$ and $\{f\xi _n\}$ are Cauchy sequences in *P*. Hence, by following the remaining proof of Theorem 2.2 of [4], we can show that *S* and *f* have a coincidence point in *P*. $\square$

### Corollary 16

*Let* $(P,d,s,\preceq )$ *be a complete partially ordered* *b*-*metric space with* $s > 1$, *and* $S:P \times P \rightarrow P$ *be a continuous mapping such that* *S* *has a mixed monotone property. Suppose there exists* $\phi \in \Phi$ *and* $\psi \in \Psi$ *such that*

$$\begin{aligned} \phi (s^kd(S(\upsilon ,\xi ),S(\rho ,\tau )))\le \phi (M_f(\upsilon ,\xi ,\rho ,\tau ))-\psi (N_f(\upsilon ,\xi ,\rho ,\tau )), \end{aligned}$$

*for all* $\upsilon ,\xi ,\rho ,\tau \in P$ *with* $\upsilon \preceq \rho$ *and* $\xi \succeq \tau$, $k>2$ *and where*

$$\begin{aligned} \begin{aligned} M_f(\upsilon ,\xi ,\rho ,\tau )&=\max \left\{ \frac{d(\rho ,S(\rho , \tau )) \left[ 1+d(\upsilon ,S(\upsilon , \xi ))\right] }{1+d(\upsilon ,\rho )}, \frac{d(\upsilon ,S(\upsilon , \xi ))\,d(\rho ,S(\rho , \tau ))}{1+d(S(\upsilon , \xi ),S(\rho , \tau ))},\right. \\&\quad \left. \frac{d(\upsilon ,S(\upsilon , \xi ))\,d(\upsilon ,S(\rho , \tau ))}{1+d(\upsilon ,S(\rho , \tau ))+d(\rho ,S(\upsilon , \xi ))}, d(\upsilon ,\rho )\right\} , \end{aligned} \end{aligned}$$

*and*

$$\begin{aligned} N_f(\upsilon ,\xi ,\rho ,\tau )=\max \left\{ \frac{d(\rho ,S(\rho ,\tau )) \left[ 1+d(\upsilon ,S(\upsilon ,\xi ))\right] }{1+d(\upsilon ,\rho )}, d(\upsilon ,\rho )\right\} . \end{aligned}$$

*Then* *S* *has a coupled fixed point in* *P*, *if there exists* $(\upsilon _0,\xi _0) \in P \times P$ *such that* $\upsilon _0 \preceq S(\upsilon _0,\xi _0)$ *and* $\xi _0 \succeq S(\xi _0,\upsilon _0)$.

### Proof

Set $f=I_P$ in Theorem 15. $\square$

### Corollary 17

*Let* $(P,d,s,\preceq )$ *be a complete partially ordered* *b*-*metric space with* $s > 1$, *and* $S:P \times P \rightarrow P$ *be a continuous mapping such that* *S* *has a mixed monotone property. Suppose there exists* $\psi \in \Psi$ *such that*

$$\begin{aligned} d(S(\upsilon ,\xi ),S(\rho ,\tau ))\le \frac{1}{s^k}M_f(\upsilon ,\xi ,\rho ,\tau )-\frac{1}{s^k}\psi (N_f(\upsilon ,\xi ,\rho ,\tau )), \end{aligned}$$

*for all* $\upsilon ,\xi ,\rho ,\tau \in P$ *with* $\upsilon \preceq \rho$ *and* $\xi \succeq \tau$, $k>2$ *where*

$$\begin{aligned} \begin{aligned} M_f(\upsilon ,\xi ,\rho ,\tau )&=\max \left\{ \frac{d(\rho ,S(\rho , \tau )) \left[ 1+d(\upsilon ,S(\upsilon , \xi ))\right] }{1+d(\upsilon ,\rho )}, \frac{d(\upsilon ,S(\upsilon , \xi ))\,d(\rho ,S(\rho , \tau ))}{1+d(S(\upsilon , \xi ),S(\rho , \tau ))},\right. \\&\quad \left. \frac{d(\upsilon ,S(\upsilon , \xi ))\,d(\upsilon ,S(\rho , \tau ))}{1+d(\upsilon ,S(\rho , \tau ))+d(\rho ,S(\upsilon , \xi ))}, d(\upsilon ,\rho )\right\} , \end{aligned} \end{aligned}$$

*and*

$$\begin{aligned} N_f(\upsilon ,\xi ,\rho ,\tau )=\max \left\{ \frac{d(\rho ,S(\rho ,\tau )) \left[ 1+d(\upsilon ,S(\upsilon ,\xi ))\right] }{1+d(\upsilon ,\rho )}, d(\upsilon ,\rho )\right\} . \end{aligned}$$

*If there exists* $(\upsilon _0,\xi _0) \in P \times P$ *such that* $\upsilon _0 \preceq S(\upsilon _0,\xi _0)$ *and* $\xi _0 \succeq S(\xi _0,\upsilon _0)$, *then* *S* *has a coupled fixed point in* *P*.

### Theorem 18

*In addition to Theorem* 15, *if for all* $(\upsilon ,\xi ),(r,s) \in P \times P$, *there exists* $(c^*,d^*)\in P \times P$ *such that* $(S(c^*,d^*), S(d^*,c^*))$ *is comparable to* $(S(\upsilon ,\xi ), S(\xi ,\upsilon ))$ *and to* (*S*(*r*, *s*), *S*(*s*, *r*)), *then* *S* *and* *f* *have a unique coupled common fixed point in* $P \times P$.

### Proof

From Theorem 15, we know that there exists atleast one coupled coincidence point in *P* for *S* and *f*. Assume that $(\upsilon , \xi )$ and (*r*, *s*) are two coupled coincidence points of *S* and *f*, i.e., $S(\upsilon , \xi )=f\upsilon$, $S(\xi ,\upsilon , )=f\xi$ and $S(r,s)=fr$, $S(s,r)=fs$. Now, we have to prove that $f\upsilon =fr$ and $f\xi =fs$.

From the hypotheses, there exists $(c^*,d^*)\in P \times P$ such that $(S(c^*,d^*), S(d^*,c^*))$ is comparable to $(S(\upsilon ,\xi ), S(\xi ,\upsilon ))$ and to (*S*(*r*, *s*), *S*(*s*, *r*)). Suppose that

$$\begin{aligned} (S(\upsilon ,\xi ), S(\xi ,\upsilon )) \le (S(c^*,d^*), S(d^*,c^*)) \,\text {and}\, (S(r,s),S(s,r))\le (S(c^*,d^*), S(d^*,c^*)). \end{aligned}$$

Let $c^*_0=c^*$ and $d^*_0=d^*$ and then choose $(c^*_1,d^*_1) \in P \times P$ as

$$\begin{aligned} fc^*_1=S(c^*_0,d^*_0),\,\, fd^*_1=S(d^*_0,c^*_0)\,\,(n \ge 1). \end{aligned}$$

By repeating the same procedure above, we can obtain two sequences $\{f c^*_{n}\}$ and $\{f d^*_{n}\}$ in *P* such that

$$\begin{aligned} fc^*_{n+1}=S(c^*_n,d^*_n),\,\, fd^*_{n+1}=S(d^*_n,c^*_n)\,\,(n \ge 0). \end{aligned}$$

Similarly, define the sequences $\{f \upsilon _{n}\}$, $\{f \xi _{n}\}$ and $\{f r_{n}\}$, $\{f s_{n}\}$ as above in *P* by setting $\upsilon _0=\upsilon$, $\xi _0=\xi$ and $r_0=r$, $s_0=s$. Further, we have that

54 $$\begin{aligned} f\upsilon _{n} \rightarrow S(\upsilon ,\xi ),\,f\xi _{n} \rightarrow S(\xi ,\upsilon ),\, fr_{n} \rightarrow S(r,s),\,fs_n \rightarrow S(s,r)\,\,(n \ge 1). \end{aligned}$$

Since, $(S(\upsilon ,\xi ), S(\xi ,\upsilon ))=(f\upsilon ,f\xi )=(f\upsilon _1,f\xi _1)$ is comparable to $(S(c^*,d^*), S(d^*,c^*))=(fc^*,fd^*)=(fc^*_1,fd^*_1)$ and hence we get $(f\upsilon _1,f\xi _1) \le (fc^*_1,fd^*_1)$. Thus, by induction we obtain that

55 $$\begin{aligned} (f\upsilon _{n},f\xi _{n}) \le (fc^*_n,fd^*_n)\,\,(n \ge 0). \end{aligned}$$

Therefore from (41), we have

56 $$\begin{aligned} \begin{aligned} \phi (d(f\upsilon ,fc^*_{n+1}))\le \phi (s^kd(f\upsilon ,fc^*_{n+1}))&= \phi (s^kd(S(\upsilon ,\xi ),S(c^*_n,d^*_n))) \\&\le \phi (M_f(\upsilon ,\xi ,c^*_n,d^*_n))-\psi (N_f(\upsilon ,\xi ,c^*_n,d^*_n)), \end{aligned} \end{aligned}$$

where

$$\begin{aligned} \begin{aligned} M_f(\upsilon ,\xi ,c^*_n,d^*_n)&=\max \left\{ \frac{d(fc^*_n,S(c^*_n, d^*_n)) \left[ 1+d(f\upsilon ,S(\upsilon , \xi ))\right] }{1+d(f\upsilon ,fc^*_n)}, \frac{d(f\upsilon ,S(\upsilon , \xi ))\,d(fc^*_n,S(c^*_n, d^*_n))}{1+d(S(\upsilon , \xi ),S(c^*_n, d^*_n))},\right. \\&\quad \left. \frac{d(f\upsilon ,S(\upsilon , \xi ))\,d(f\upsilon ,S(c^*_n, d^*_n))}{1+d(f\upsilon ,S(c^*_n, d^*_n))+d(fc^*_n,S(\upsilon , \xi ))}, d(f\upsilon ,fc^*_n)\right\} \\&= \max \{0,0,0,d(f\upsilon ,fc^*_n)\} \\&=d(f\upsilon ,fc^*_n) \end{aligned} \end{aligned}$$

and

$$\begin{aligned} \begin{aligned} N_f(\upsilon ,\xi ,c^*_n,d^*_n)&=\max \left\{ \frac{d(fc^*_n,S(c^*_n,d^*_n)) \left[ 1+d(f\upsilon ,S(\upsilon ,\xi ))\right] }{1+d(f\upsilon ,fc^*_n)}, d(f\upsilon ,fc^*_n)\right\} \\&=d(f\upsilon ,fc^*_n). \end{aligned} \end{aligned}$$

Thus from (56),

57 $$\begin{aligned} \phi (d(f\upsilon ,fc^*_{n+1}))\le \phi (d(f\upsilon ,fc^*_n))-\psi (d(f\upsilon ,fc^*_n)). \end{aligned}$$

As by the similar process, we can prove that

58 $$\begin{aligned} \phi (d(f\xi ,fd^*_{n+1}))\le \phi (d(f\xi ,fd^*_n))-\psi (d(f\xi ,fd^*_n)). \end{aligned}$$

From (57) and (58), we have

59 $$\begin{aligned} \begin{aligned} \phi (\max \{d(f\upsilon ,fc^*_{n+1}),d(f\xi ,fd^*_{n+1})\})&\le \phi (\max \{d(f\upsilon ,fc^*_n),d(f\xi ,fd^*_n)\})\\&\quad -\psi (\max \{d(f\upsilon ,fc^*_n),d(f\xi ,fd^*_n)\}) \\&<\phi (\max \{d(f\upsilon ,fc^*_n),d(f\xi ,fd^*_n)\}). \end{aligned} \end{aligned}$$

Hence by the property of $\phi$, we get

$$\begin{aligned} \max \{d(f\upsilon ,fc^*_{n+1}),d(f\xi ,fd^*_{n+1})\} <\max \{d(f\upsilon ,fc^*_n),d(f\xi ,fd^*_n)\}, \end{aligned}$$

which shows that $\max \{d(f\upsilon ,fc^*_n),d(f\xi ,fd^*_n)\}$ is a decreasing sequence and by a result there exists $\gamma \ge 0$ such that

$$\begin{aligned} \lim \limits _{n \rightarrow +\infty }\max \{d(f\upsilon ,fc^*_n),d(f\xi ,fd^*_n)\} =\gamma . \end{aligned}$$

From (59) taking upper limit as $n \rightarrow +\infty$, we get

60 $$\begin{aligned} \phi (\gamma )\le \phi (\gamma )-\psi (\gamma ), \end{aligned}$$

from which we get $\psi (\gamma )=0$, implies that $\gamma =0$. Thus,

$$\begin{aligned} \lim \limits _{n \rightarrow +\infty }\max \{d(f\upsilon ,fc^*_n),d(f\xi ,fd^*_n)\} =0. \end{aligned}$$

Consequently, we get

61 $$\begin{aligned} \lim \limits _{n \rightarrow +\infty }d(f\upsilon ,fc^*_n) =0 \, \text {and} \,\lim \limits _{n \rightarrow +\infty }d(f\xi ,fd^*_n) =0. \end{aligned}$$

By similar argument, we get

62 $$\begin{aligned} \lim \limits _{n \rightarrow +\infty }d(fr,fc^*_n) =0 \, \text {and} \,\lim \limits _{n \rightarrow +\infty }d(fs,fd^*_n) =0. \end{aligned}$$

Therefore from (61) and (62), we get $f\upsilon =fr$ and $f\xi =fs$. Since $f\upsilon =S(\upsilon ,\xi )$ and $f\xi =S(\xi ,\upsilon )$, then by the commutativity of *S* and *f*, we have

63 $$\begin{aligned} f(f\upsilon )= f(S(\upsilon ,\xi ))=S(f\upsilon ,f\xi )\, \text {and}\,f(f\xi )= f(S(\xi ,\upsilon ))=S(f\xi ,f\upsilon ). \end{aligned}$$

Let $f\upsilon =a^*$ and $f\xi =b^*$ then (63) becomes

64 $$\begin{aligned} f(a^*)= S(a^*,b^*)\, \text {and}\,f(b^*)= S(b^*,a^*), \end{aligned}$$

which shows that $(a^*,b^*)$ is a coupled coincidence point of *S* and *f*. It follows that $f(a^*)=fr$ and $f(b^*)=fs$ that is $f(a^*)=a^*$ and $f(b^*)=b^*$. Thus from (64), we get $a^*=f(a^*)= S(a^*,b^*)$ and $b^*=f(b^*)= S(b^*,a^*)$. Therefore, $(a^*,b^*)$ is a coupled common fixed point of *S* and *f*.

For the uniqueness let $(u^*,v^*)$ be another coupled common fixed point of *S* and *f*, then we have $u^*=fu^*= S(u^*,v^*)$ and $v^*=fv^*= S(v^*,u^*)$. Since $(u^*,v^*)$ is a coupled common fixed point of *S* and *f*, then we obtain $fu^*=f\upsilon =a^*$ and $fv^*=f\xi =b^*$. Thus, $u^*=fu^*=fa^*=a^*$ and $v^*=fv^*=fb^*=b^*$. Hence the result. $\square$

### Theorem 19

*In addition to the hypotheses of Theorem* 18, *if* $f\upsilon _0$ *and* $f\xi _0$ *are comparable, then* *S* *and* *f* *have a unique common fixed point in* *P*.

### Proof

From Theorem 18, *S* and *f* have a unique coupled common fixed point $(\upsilon ,\xi ) \in P$. Now, it is enough to prove that $\upsilon =\xi$. From the hypotheses, we have $f\upsilon _0$ and $f\xi _0$ are comparable then we assume that $f\upsilon _0 \preceq f\xi _0$. Hence by induction we get $f\upsilon _n \preceq f\xi _n$ for all $n \ge 0$, where $\{f\upsilon _n\}$ and $\{f\xi _n\}$ are from Theorem 15.

Now by use of Lemma 6, we get

$$\begin{aligned} \begin{aligned} \phi (s^{k-2}d(\upsilon ,\xi ))&=\phi (s^k \frac{1}{s^2}d(\upsilon ,\xi )) \le \lim \limits _{n \rightarrow +\infty }\sup \phi (s^k d(\upsilon _{n+1}, \xi _{n+1})) \\&= \lim \limits _{n \rightarrow +\infty }\sup \phi (s^k d(S(\upsilon _n, \xi _n),S(\xi _n,\upsilon _n))) \\&\le \lim \limits _{n \rightarrow +\infty }\sup \phi (M_f(\upsilon _n, \xi _n,\xi _n,\upsilon _n))-\lim \limits _{n \rightarrow +\infty }\inf \psi (N_f(\upsilon _n, \xi _n,\xi _n,\upsilon _n)) \\&\le \phi (d(\upsilon ,\xi ))-\lim \limits _{n \rightarrow +\infty }\inf \psi (N_f(\upsilon _n, \xi _n,\xi _n,\upsilon _n)) \\&<\phi (d(\upsilon ,\xi )), \end{aligned} \end{aligned}$$

which is a contradiction. Thus, $\upsilon =\xi$, i.e., *S* and *f* have a common fixed point in *P*. $\square$

### Remark 20

It is well known that *b*-metric space is a metric space when $s=1$. So, from the result of Jachymski [39], the condition

$$\begin{aligned} \phi (d(S(\upsilon ,\xi ),S(\rho ,\tau ))) \le \phi (\max \{d(f\upsilon ,f\rho ),d(f\xi ,f\tau )\})-\psi (\max \{d(f\upsilon ,f\rho ),d(f\xi ,f\tau )\}) \end{aligned}$$

is equivalent to,

$$\begin{aligned} d(S(\upsilon ,\xi ),S(\rho ,\tau ))\le \varphi (\max \{d(f\upsilon ,f\rho ),d(f\xi ,f\tau )\}), \end{aligned}$$

where $\phi \in \Phi$, $\psi \in \Psi$ and $\varphi :[0,+\infty )\rightarrow [0,+\infty )$ is continuous, $\varphi (t)<t$ for all $t>0$ and $\varphi (t)=0$ if and only if $t=0$. So, in view of above our results generalize and extend the results of [19, 20, 40–42] and several other comparable results.

### Corollary 21

*Suppose* $(P,d,s,\preceq )$ *be a complete partially ordered* *b*-*metric space with parameter* $s > 1$. *Let* $S:P \rightarrow P$ *be a continuous, nondecreasing map with regards to* $\preceq$ *such that there exists* $\upsilon _0 \in P$ *with* $\upsilon _0 \preceq S\upsilon _0$. *Suppose that*

65 $$\begin{aligned} \phi (sd(S\upsilon ,S\xi ))\le \phi (M(\upsilon ,\xi ))-\psi (M(\upsilon ,\xi )), \end{aligned}$$

*where* $M(\upsilon ,\xi )$ *and the conditions upon* $\phi , \psi$ *are same as in Theorem* 8. *Then* *S* *has a fixed point in* *P*.

### Proof

Set $N(\upsilon ,\xi )=M(\upsilon ,\xi )$ in a contraction condition (3) and apply Theorem 8, we have the required proof. $\square$

### Note 22

Similarly by removing the continuity of a nondecreasing mapping *S* and taking a nondecreasing sequence $\{\upsilon _n\}$ as above in Theorem 9, we can obtain a fixed point for *S* in *P*. Also one can obtains the uniqueness of a fixed point of *S* by using condition (17) in *P* and following the proof of Theorem 10.

### Note 23

By following the proofs of Theorem 12 and 13, we can find the coincidence point for *S* and *f* in *P*. Similarly, from Theorem 15, 18 and 19, one can obtain a coupled coincidence point and its uniqueness, and a unique common fixed point for mappings *S* and *f* in $P\times P$ satisfying an almost generalized contraction condition (65), where $M_f(\upsilon ,\xi )$, $M_f(\upsilon ,\xi , \rho , \tau )$ and the conditions upon $\phi , \psi$ are same as above defined.

### Corollary 24

*Suppose that* $(P,d,s,\preceq )$ *be a complete partially ordered* *b*-*metric space with* $s > 1$. *Let* $S:P \rightarrow P$ *be a continuous, nondecreasing mapping with regards to* $\preceq$. *If there exists* $k \in [0,1)$ *and for any* $\upsilon ,\xi \in P$ *with* $\upsilon \preceq \xi$ *such that*

66 $$\begin{aligned} \begin{aligned} d(S\upsilon ,S\xi )&\le \frac{k}{s}\max \left\{ \frac{d(\xi ,S\xi ) \left[ 1+d(\upsilon ,S\upsilon )\right] }{1+d(\upsilon ,\xi )}, \frac{d(\upsilon ,S\upsilon )\,d(\xi ,S\xi )}{1+d(S\upsilon ,S\xi )},\right. \\&\quad \left. \frac{d(\upsilon ,S\upsilon )\,d(\upsilon ,S\xi )}{1+d(\upsilon ,S\xi )+d(\xi ,S\upsilon )}, d(\upsilon ,\xi )\right\} . \end{aligned} \end{aligned}$$

*If there exists* $\upsilon _0 \in P$ *with* $\upsilon _0 \preceq S\upsilon _0$, *then* *S* *has a fixed point in* *P*.

### Proof

Set $\phi (t)=t$ and $\psi (t)=(1-k)t$, for all $t \in (0, +\infty )$ in Corollary 21. $\square$

### Note 25

Relaxing the continuity of a map *S* in Corollary 24, one can obtains a fixed point for *S* on taking a nondecreasing sequence $\{\upsilon _n\}$ in *P* by following the proof of Theorem 9.

We illustrate the usefulness of the obtained results in different cases such as continuity and discontinuity of a metric *d* in a space *P*.

### Example 26

Define a metric $d:P \rightarrow P$ as below and $\le$ is an usual order on *P*, where $P=\{1,2,3,4,5,6\}$

$$ \begin{gathered}   d(\upsilon ,\xi ) = d(\upsilon ,\xi ) = 0,\quad if\;\upsilon ,\xi  = 1,2,3,4,5,6\;{\text{and}}\;\upsilon  = \xi , \hfill \\   d(\upsilon ,\xi ) = d(\upsilon ,\xi ) = 3,\quad if\;\upsilon ,\xi  = 1,2,3,4,5\;{\text{and}}\;\upsilon  \ne \xi , \hfill \\   d(\upsilon ,\xi ) = d(\upsilon ,\xi ) = 12,\quad if\;\upsilon  = 1,2,3,4\;{\text{and}}\;\upsilon  = 6, \hfill \\   d(\upsilon ,\xi ) = d(\upsilon ,\xi ) = 20,\quad if\;\upsilon  = 5\;{\text{and}}\;\xi  = 6. \hfill \\  \end{gathered}  $$

Define a map $S:P \rightarrow P$ by $S1=S2=S3=S4=S5=1, S6=2$ and let $\phi (t)=\frac{t}{2}$, $\psi (t)=\frac{t}{4}$ for $t \in [0,+\infty )$. Then *S* has a fixed point in *P*.

### Proof

It is apparent that, $(P,d,s,\preceq )$ is a complete partially ordered *b*-metric space for $s=2$. Consider the possible cases for $\upsilon$, $\xi$ in *P*:

**Case 1**. Suppose $\upsilon , \xi \in \{1,2,3,4,5\}$, $\upsilon <\xi$ then $d(S\upsilon ,S\xi )=d(1,1)=0$. Hence,

$$\begin{aligned} \phi (2d(S\upsilon ,S\xi ))=0 \le \phi (M(\upsilon ,\xi ))-\psi (M(\upsilon ,\xi )). \end{aligned}$$

**Case 2**. Suppose that $\upsilon \in \{1,2,3,4,5\}$ and $\xi =6$, then $d(S\upsilon ,S\xi )=d(1,2)=3$, $M(6,5)=20$ and $M(\upsilon ,6)=12$, for $\upsilon \in \{1,2,3,4\}$. Therefore, we have the following inequality,

$$\begin{aligned} \phi (2d(S\upsilon ,S\xi )) \le \frac{M(\upsilon ,\xi )}{4} =\phi (M(\upsilon ,\xi ))-\psi (M(\upsilon ,\xi )). \end{aligned}$$

Thus, condition (65) of Corollary 21 holds. Furthermore, the remaining assumptions in Corollary 21 are fulfilled. Hence, *S* has a fixed point in *P* as Corollary 21 is appropriate to $S, \phi , \psi$ and $(P,d,s,\preceq )$. $\square$

### Example 27

A metric $d:P \rightarrow P$, where $P=\{0, 1, \frac{1}{2},\frac{1}{3},\frac{1}{4},........\frac{1}{n},.....\}$ with usual order $\le$ is as follows

$$\begin{aligned} \begin{aligned} d(\upsilon ,\xi )= {\left\{ \begin{array}{ll} &{} \,0 \,\,\,\,\,\,\,\,\,,\,\, if\, \upsilon =\xi \\ &{} \,1 \,\,\,\,\,\,\,\,\,,\,\, if\, \upsilon \ne \xi \in \{0,1\} \\ &{} \,|\upsilon -\xi |\,\,\,,\,\, if\, \upsilon ,\xi \in \left\{ 0, \frac{1}{2n},\frac{1}{2m}: n \ne m \ge 1\right\} \\ &{} \,6 \,\,\,\,\,\,\,\,\,\,,\,\, otherwise. \end{array}\right. } \end{aligned} \end{aligned}$$

A map $S: P \rightarrow P$ be such that $S0=0, S\frac{1}{n}=\frac{1}{12n}$ for all $n\ge 1$ and let $\phi (t)=t$, $\psi (t)=\frac{4t}{5}$ for $t \in [0,+\infty )$. Then, *S* has a fixed point in *P*.

### Proof

It is obvious that for $s=\frac{12}{5}$, $(P,d,s,\preceq )$ is a complete partially ordered *b*-metric space and also by definition, *d* is discontinuous *b*-metric space. Now for $\upsilon ,\xi \in P$ with $\upsilon <\xi$, then we have the following cases:

**Case 1**. If $\upsilon =0$ and $\xi =\frac{1}{n}$, $n \ge 1$, then $d(S\upsilon ,S\xi )=d(0,\frac{1}{12n})=\frac{1}{12n}$ and $M(\upsilon ,\xi )=\frac{1}{n}$ or $M(\upsilon ,\xi )= \{1,6\}$. Therefore, we have

$$\begin{aligned} \phi \left( \frac{12}{5}d(S\upsilon ,S\xi )\right) \le \frac{M(\upsilon ,\xi )}{5} =\phi (M(\upsilon ,\xi ))-\psi (M(\upsilon ,\xi )). \end{aligned}$$

**Case 2**. If $\upsilon =\frac{1}{m}$ and $\xi =\frac{1}{n}$ with $m>n\ge 1$, then

$$\begin{aligned} d(S\upsilon ,S\xi )=d(\frac{1}{12m},\frac{1}{12n}) \,\text {and}\, M(\upsilon ,\xi )\ge \frac{1}{n}-\frac{1}{m}\, \text {or}\, M(\upsilon ,\xi )=6. \end{aligned}$$

Therefore,

$$\begin{aligned} \phi \left( \frac{12}{5}d(S\upsilon ,S\xi )\right) \le \frac{M(\upsilon ,\xi )}{5} =\phi (M(\upsilon ,\xi ))-\psi (M(\upsilon ,\xi )). \end{aligned}$$

Hence, condition (65) of Corollary 21 and remaining assumptions are satisfied. Thus, *S* has a fixed point in *P*. $\square$

### Example 28

Let $P=C[a,b]$ be the set of all continuous functions. Let us define a *b*-metric *d* on *P* by

$$\begin{aligned} d(\theta _1,\theta _2)=\sup _{t \in C[a,b]}\{|\theta _1(t)-\theta _2(t)|^2\} \end{aligned}$$

for all $\theta _1,\theta _2 \in P$ with partial order $\preceq$ defined by $\theta _1 \preceq \theta _2$ if $a\le \theta _1(t) \le \theta _2 (t)\le b$, for all $t \in [a,b]$, $0 \le a<b$. Let $S:P \rightarrow P$ be a mapping defined by $S \theta = \frac{\theta }{5}, \theta \in P$ and the two altering distance functions by $\phi (t)=t$, $\psi (t)=\frac{t}{3}$, for any $t \in [0, +\infty ]$. Then *S* has a unique fixed point in *P*.

### Proof

By the hypotheses, it is clear that $(P,d,s,\preceq )$ is a complete partially ordered *b*-metric space with parameter $s=2$ and fulfill all conditions of Corollary 21 and Note 22. Furthermore for any $\theta _1,\theta _2 \in P$, the function $\min (\theta _1,\theta _2) (t)=\min \{\theta _1(t),\theta _2(t)\}$ is also continuous and the conditions of Corollary 21 and Note 22 are satisfied. Hence, *S* has a unique fixed point $\theta =0$ in *P*. $\square$

## Limitations

In this manuscript, some fixed point, coincidence point, coupled coincidence point and coupled common fixed point results for mappings satisfying generalized $(\phi , \psi )$-contraction conditions in complete partially ordered *b*-metric spaces are proved. These results generalize and extend some known results in the existing literature. Few examples are presented at the end to support our results.

## Data Availability

Not applicable.
